# Collating existing evidence on cumulative impacts of invasive plant species in riparian ecosystems of British Columbia, Canada: a systematic map protocol

**DOI:** 10.1186/s13750-023-00320-3

**Published:** 2023-12-14

**Authors:** Fabio Mologni, Chandra E. Moffat, Jason Pither

**Affiliations:** 1 Department of Biology, and Institute for Biodiversity, Resilience, and Ecosystem Services, I.K. Barber Faculty of Science, University of British Columbia Okanagan, 1177 Research Road, Kelowna, BC V1V 1V7 Canada; 2 Agriculture and Agri-Food Canada, Summerland Research and Development Centre, 4200 Highway #97, Summerland, BC V0H 1Z0 Canada

**Keywords:** Cumulative impacts, British Columbia, Invasive species, Impacts, Riparian ecosystems, Plant invasions, Foreshore ecosystems, Protocol, Systematic maps

## Abstract

**Background:**

Globally, the structure and functioning of foreshore and riparian ecosystems are being dramatically impacted by non-native invasive plant species. Invasive species can outcompete and replace native species, modify geochemical and hydraulic cycles, alter trophic processes, and change the composition and structure of communities above and below ground. However, these impacts are often investigated in isolation, even though one invasive species might increase or mitigate the impacts of others (i.e. cumulative impacts), potentially with cascading effects. Although cumulative impacts have long been studied within other environmental contexts, research on the cumulative impacts of invasive species is comparatively scarce. We aim to develop a protocol to systematically identify and collate evidence on the individual and cumulative impacts of a set of plant species invasive in foreshore and riparian ecosystems of British Columbia, Canada. Our primary question is: what evidence is available on the individual and cumulative impacts of invasive plants in the riparian and foreshore ecosystems of British Columbia, Canada? In addition, our systematic map will identify the strengths and gaps in knowledge pertaining to invasive plant species impacts in foreshore and riparian ecosystems, with the ultimate goal of facilitating the development of evidence-based management strategies.

**Methods:**

We identified the research topic and the primary and secondary questions with the support of stakeholders. We then devised a flexible string that allows for searching target invasive species. Using this string, we searched the literature for pilot species that aided the iterative development of the protocol. Once all target species are identified, we will carry out a systematic literature search on their impacts. We will search Web of Science and the CABI compendium for invasive species. We will include studies if they (i) refer to the target invasive species, (ii) focus on its environmental impacts and (iii) investigate such impacts in riparian ecosystems (iv) within North America (i.e. Canada and U.S.A.). We will use a two-stage screening process: titles and abstracts first, then the full manuscript. From each source, we will extract impact description, ecosystem component impacted, and magnitude and directionality of impacts. We will include a publicly available database of studies, descriptive statistics, and a narrative summary within our synthesis outcomes.

**Supplementary Information:**

The online version contains supplementary material available at 10.1186/s13750-023-00320-3.

## Background

### Biological invasions in foreshore and riparian ecosystems

Foreshore and riparian ecosystems are vitally important from ecological, cultural, and economic standpoints. Although their spatial extent is small, they are often hotspots of biodiversity, hosting rare species, and serving as refugia and corridors essential to many others [1–3]. These ecosystems also provide essential functions and services such as improving water quality, flood mitigation, and minimizing erosion [2, 4, 5]. As such, foreshore and riparian habitats are the focus of targeted management and conservation strategies in many countries [6–9].

Despite their recognized importance, foreshore and riparian ecosystems are being impacted by many anthropogenic stressors [10]. Infrastructures (e.g. dams, dyking, channelization) and water management (e.g. water diversion, irrigation, dredging) can radically modify water levels and flow and disrupt natural fluvial dynamics [1, 5, 11, 12]. Contamination and nutrient additions can alter water quality, reduce biodiversity, and promote bioaccumulation [1, 13]. Habitat loss through agriculture, deforestation, and development disproportionately impacts foreshore and riparian zones [1, 14–16], and was estimated to be up to two-thirds in the U.S. alone [17]. Additionally, freshwater ecosystems are oftentimes highly invaded by non-native species due to their proximity to human settlements and their function as dispersal corridors [14, 18–21].

Invasive species can impact riparian ecosystems in various ways, but invasive plants have particularly pervasive impacts on ecosystem structure and functioning. By spreading aggressively, they displace both plant and animal native species [22–25], modify geochemical and hydraulic cycles [26, 27], alter trophic processes [28], and change the composition and structure of communities above and below ground [2, 29]. Additionally, invasive plants alter traditional practices and resource use by Indigenous peoples [28]. The cumulative impacts of invasive plants on riparian ecosystems are potentially profound, but research to quantify such effects remains limited [2, 30].

Here, we aim to develop a framework for systematically collating and mapping evidence on the individual and cumulative impacts of plant species that are invasive within foreshore and riparian ecosystems, and we will apply our protocol to systems in British Columbia, Canada.

### Individual and cumulative impacts: definitions, examples and previous work

In invasion ecology, individual impacts are defined as measurable changes caused by non-native species on a target ecosystem [31, 32]. They can vary greatly in type, magnitude, and directionality. For instance, some impacts might be barely detectable (e.g. gene flow through hybridization), while others can produce pronounced, observable effects (e.g. ecosystem dominance). Impacts can be direct (e.g. displacement of native species), but also mediated through other factors (e.g. competition for resources, [31]). Finally, while non-native species have been investigated in large part because of their negative effects, impacts can vary along a continuum from negative to positive [32, 33], and can be ecosystem or context-dependent.

Identifying an impact’s directionality presents some challenges. Negative impacts are typically equated to unfavourable outcomes for humans [32]. However, this approach is strongly biased by the value system and worldview of the researcher [33, 34]. In an effort to minimize subjectivity and value-based identifications of impact directionality, we define as negative or positive any quantifiable reduction or increase in ecosystem properties or attributes (e.g. native species richness and abundance, nutrient cycling, water quality, etc., [32]). For instance, we define as positive an increase in the fitness or number of individuals of a native species but as negative its reduction.

The combination and interaction of multiple individual impacts are referred to as cumulative impacts and many definitions of this concept exist. For the Canadian Environmental Assessment Act (CEAA), they are *“changes to the environment that are caused by an action in combination with other past, present and future human actions*” [35]. The Council on Environmental Quality (CEQ) suggests impacts have to be incremental [36]. The most well-articulated definition is that of the European Environmental Agency (EEA), which defines them as: ‘*the impacts (positive or negative, direct and indirect, long-term and short-term impacts) arising from a range of activities throughout an area or region, where each individual effect may not be significant if taken in isolation. Such impacts can arise from the growing volume of traffic, the combined effect of a number of agriculture measures leading to more intensive production and use of chemicals, *etc*. Cumulative impacts include a time dimension, since they should calculate the impact on environmental resources resulting from changes brought about by past, present and reasonably foreseeable future actions.”* [37]. Consistent elements among these definitions are (1) the combination of multiple individual impacts, (2) a time component and (3) the human agency. While not explicitly stated in the previous definitions, cumulative impacts also have a spatial dimension, or they can accumulate in space as well as temporally [38].

We define cumulative impacts in biological invasions as the combined effect of multiple impacts when at least one is generated by an invasive species. Cumulative impacts include recurrent impacts of a single species and the combined effect of multiple invaders, but also the compounded impact of invading species and other anthropogenic stressors [12]. Our definition incorporates all the elements of previous definitions; however, it is more restrictive, as the primary focus is the impacts of invasive species. Conversely, it includes impacts of any magnitude, type or directionality.

The term ‘cumulative’ might imply that the total effect of multiple impacts is always greater than that of individual impacts. Multiple invaders can collectively increase native species displacement, or enhance topsoil nutrient concentration (additive impacts, [29, 39]). An N-fixer might increase soil nitrogen, facilitating invasions by more competitive nitrophilous species, which in turn will displace natives (multiplicative impacts, [29]). However, additive or multiplicative impacts are not the only potential outcomes. Competition between two invaders might instead reduce their impact per capita. For example, an allopathic species might negatively affect both native and non-native species. In this case, one invader mitigates the impacts of another invader [38].

Despite a long history of research on cumulative impacts within environmental contexts [38], the literature on the cumulative impacts of invasive species is relatively scarce. Most work in biological invasions focuses on a single species or single direct impact [40–45]. Even when multiple impacts are identified, their cumulative effect is rarely considered [30, 46]. This is despite previously proposed theoretical frameworks share some conceptual overlap. One such example is the invasion meltdown, which posits that interactions among invaders might increase their impacts [47]. Critically for our work, little research effort explored the cumulative impacts of invasive plant species in riparian and foreshore ecosystems. Therefore, anticipating a lack of studies on cumulative impacts, we will also include individual impacts in this systematic map.

### Topic identification and stakeholder input

There is a clear need for work identifying the cumulative impacts of invasive species in riparian ecosystems. The Province of British Columbia, Ministry of Forests Invasive Plant Program, highlighted the need to synthesize current evidence on the impacts of invasive plant species in riparian and foreshore ecosystems within the province, to inform research and management needs. British Columbia’s riparian and foreshore ecosystems are invaded by numerous highly destructive invasive plant species, such as Russian Olive (*Elaeagnus angustifolia*), Phragmites (*Phragmites australis*), Knotweeds (*Reynoutria* spp., syn. *Fallopia*), Tree of Heaven (*Ailanthus altissima*) and Canary reed grass (*Phalaris arundinacea*). While the impacts of these species have been extensively investigated [42, 48–52], there is no comprehensive assessment of their cumulative impacts.

Stakeholders in the provincial government played a pivotal role in shaping the research topic and refining the scope of the systematic map. Stakeholders include the British Columbia Ministry of Forests, Agriculture and Agri-Food Canada, and the University of British Columbia. Based on their expert knowledge and the available data, they provided a list of 10–15 plant species that are invasive in the target ecosystems and geographic areas, thereby aiding in the identification of specific research questions and objectives. Input from practitioners and other researchers helped refine the approach and the methodology. Through ongoing dialogue and feedback, stakeholders were able to establish clear expectations, develop a robust methodology, and identify appropriate outcomes for the systematic map. In addition to quantifying the cumulative impacts of plant species invasive to riparian ecosystems, stakeholders have identified two additional aspects as essential. First is the development of a reproducible protocol that can be employed in future systematic studies of invasive species impacts. Second is the investigation of how the cumulative impacts of invasive species will vary under current climate change scenarios.

Protocols are a crucial aspect of developing a project, particularly in the case of systematic work [53]. Good protocols need to be transparent, detailed and reproducible, allowing other researchers to replicate their work [53–56]. In this case, we do not simply want to describe our procedure for mapping the existing literature, but we specifically aim to provide a tool that is sufficiently flexible and reproducible to be applied in the investigation of other invasive species or ecosystems.

Climate change is a key contributor to the cumulative impacts of invasive species across both terrestrial and aquatic ecosystems. However, the nature and magnitude of its effect of invasive species’ impacts is often unclear. Interactions between particular invasive plants and the diverse facets of climate change are challenging to predict and likely species- and context-dependent [57]. For instance, while the ranges of many non-native invasive species may expand as temperature rises [58], others may contract or shift in response to both abiotic and biotic factors [57, 59]. Nevertheless, strategies for mitigating negative impacts are sorely needed. A key first step is synthesizing the diverse and extensive research on this topic.

Here, we present a reproducible systematic map protocol [53] for screening, collating, and describing research on the impacts of priority invasive plants in riparian and foreshore ecosystems, and we will apply it to systems in British Columbia. Given their efficacy and comprehensiveness, systematic maps are increasingly common in environmental management [54]. Through the systematic map process, we will identify knowledge clusters and gaps (i.e. areas of high and low concentration of the research effort), and synthesize results within the context of current climate change scenarios. Key outputs will include (1) a robust analytical framework for qualitatively predicting—based on the best available evidence—the cumulative impacts of invasive plants under changing climates and followed by (2) a more detailed assessment for a selection of priority invasive plant species (identified by the BC Ministry of Forests Invasive Alien Plant Program). These outputs will have high utility for policy, planning and strategic, evidence-based decision management of ecosystems impacted by priority invasive plant species in British Columbia.

## Objective of the review

We aim to systematically collate and map evidence on the individual and cumulative impacts of a selection of plant species invasive to riparian ecosystems in British Columbia, Canada.

### Primary question

What evidence is available on the individual and cumulative impacts of invasive plants in the riparian and foreshore ecosystems of British Columbia, Canada?

### Components of the primary question

- Population: Riparian and foreshore ecosystems in British Columbia
- Exposure: non-native plant species invasive to riparian and foreshore ecosystems of British Columbia
- Comparator: No impact or absence of invasive plant species.
- Outcome: A synthesis of both the individual and collective cumulative impacts of the selected invasive plant species

### Secondary question

We will describe variations in the research effort with regard to:

- Geography and fluvial systems investigated
- Invasive species
- Impacts and their directionality (negative, positive, or neutral)
- Impacted ecosystem components
- Type of study (e.g. correlational, experimental, etc.)
- Time (did the level of knowledge change over time?)

Additionally, we will delineate potential changes in impact magnitude by species under current climate change scenarios based on the available literature.

## Methods

### Search string

We will conduct multiple systematic searches, one for each of our focus species. For each search, we will use as keywords the scientific name of a species and “impact”, formatted for Web of Science (WOS). For example:

*Elaeagnus angustifolia* AND impact*

The selected search string is purposely broad. Searches including keywords associated with the target ecosystem (riparian, foreshore, freshwater, wetland, aquatic, etc.) and geographic area (British Columbia, Canada, North America, etc.) were deemed to be too restrictive. A broader search allows for capturing additional studies that either use different keywords or investigate impacts in different circumstances and yet might be relevant to the target ecosystem.

We tested the comprehensiveness of searches using two pilot species, the Russian Olive (*Elaeagnus angustifolia*) and the Canary Reed Grass (*Phalaris arundinacea*). For each species, we selected 5 primary articles, which used a variety of keywords (e.g. impact, effect, alter, change, consequence, see Additional file 1: Appendix 1 for the full list). Then, we used the search strings to extract studies from WOS and we extracted references from CABI and review studies for pilot species. All studies were detected by search strings. These two species aided the iterative development of the protocol and will be included in the systematic map.

### Bibliographic sources

We will conduct searches in WOS, accessing the core database using an institutional licence (University of British Columbia). The core database assigns metadata to a study based exclusively on the information provided by the publisher and journal. Since other databases assign additional metadata to a study, some material might go undetected despite meeting our criteria. We will expand our search to all databases and then refine it to the core collection. This will identify studies that match our keywords across all databases but are only present in the core collection, and thus accessible to the authors (Mathew Vis-Dunbar, UBC librarian, pers. comm. 2023). Additionally, we will screen all references in the CABI Invasive Species Compendium factsheet for each species, except for references in the *Distribution References* section. Review studies that fit the criteria for inclusion will be used as sources as well, and references extracted and screened.

We will also scope organization websites across North America at different administrative levels. We will assess international (outside Canada), federal (Canada), provincial (British Columbia) and local (regions within British Columbia) organizations. We will search for the focus species name and the word “invasive” in the following organization websites:

- Canadian Weed Society
- British Columbia Inter-Ministry Invasive Species Working Group
- Canadian Council of Invasive Species
- Invasive Species Centre
- Okanagan Basin Waterboard
- North American Invasive Species Management Association (NAISMA)
- The National Environmental Coalition on Invasive Species (NECIS)
- United States Department of Agriculture (USDA)
- National Invasive Species Council
- All local associations in British Columbia (e.g. Boundary Invasive Species Society, East Kootenay Invasive Species Council, Okanagan and Similkameen Invasive Species Society, etc.)

We will conduct the same query in the following searchable catalogues of government documents:

- Canadian Federal Science Library Network
- Legislative library of British Columbia

These sources will allow for capturing also the grey literature. WOS identifies dissertations and conference proceedings, especially if expanding searches to all databases, while the CABI, review papers and organizational websites will identify technical reports.

Accessing multiple databases will help reduce location and index biases (i.e. not all journals are indexed in all databases, incomplete or poor indexing, [45]).

### Screening and inclusion criteria

The screening process will include two stages. First, we will screen titles and abstracts. If the information is insufficient to make a decision, we will assess the full manuscript as well. These steps will be applied to all studies, regardless of the source they were extracted from. A single reviewer will conduct the screening (FM). A random subset of studies will also be assessed by a second reviewer (JP) at both stages (Stage 1 = 5%, Stage 2 = 10%). We will appraise consistency using Cohen’s kappa statistics and set 0.6 as a threshold [60, 61]. If consistency is below the cut-off limit, screening and inclusion criteria will be adjusted for clarity. All disagreements will be discussed and resolved. Any study authored by one of the systematic reviewers that meets the criteria for inclusion will be assessed by the other reviewer at every stage of the process.

We will screen both commercially published and grey literature, but not personal communications or expert opinions. Including grey literature reduces the risk of publication and citation biases (i.e. significant results are more likely to be published and cited than non-significant results, [45, 48]). We will consider only material in English. To minimize language bias (i.e. significant results are more likely to be published in English, [45, 48]), we will assess the title and abstract if translated into English. Studies will be included irrespective of the magnitude, type or directionality of the impact (negative, positive or neutral), and irrespective of the statistical significance of reported results. This will help reduce the prevailing paradigm bias (i.e. a bias towards studies supporting the prevailing paradigm; in this case, invasive species’ impacts are extensive and negative, [26, 45, 48]). The time span includes all studies up to the day the search will be conducted, countering temporal bias (i.e. older studies might be overlooked, [45, 54]). Finally, we will include studies regardless of study design (e.g. experimental, observational, etc.).

We will include studies if they:

1. Refer to the non-native invasive plant species searched. We defined as invasive widespread, impactful non-native species.
2. Focus on its abiotic and biotic impacts. We defined impacts as measurable changes caused by non-native species on a target ecosystem.
3. Investigate such impacts in riparian and foreshore ecosystems. Riparian ecosystems are defined as areas adjacent to streams or rivers (flowing water), while foreshore ecosystems are defined as the land adjacent to still (non-flowing) water bodies.
4. Within North America (i.e. Canada and U.S.A.).

We will include all studies in North America because many environmental conditions and invasive species will be shared between British Columbia and other regions within Canada and the U.S. However, including all studies in North America might capture information not relevant to British Columbia. For instance, studies might investigate the impacts of invasive plant species on abiotic and biotic components absent in our study system. Such cases will be excluded, and exclusions justified. Similarly, we will justify all other exceptions [62].

### Study validity assessment

We assessed the validity of each study based only on the eligibility criteria.

### Data coding

For each study at the full-text screening stage, we will provide the following information:

1. Bibliographic information
  - Authors list
  - Article title
  - Publication year
  - Bibliographic source
2. Inclusion criteria
  - Exposure: Focuses on target species (Y/N)
  - Exposure: Focuses on abiotic and biotic impacts (Y/N)
  - Population: Focuses on riparian and foreshore ecosystems (Y/N)
  - Population: Within North America (Y/N)
3. Screening stage
  - Excluded at full-text stage
  - Included
  - Exceptions
4. Additional information
  - Duplicate (Y/N)
  - Notes

For included studies only, we will provide also the following information:

1. Bibliographic information
  - Authors list
  - Article title
  - Publication year
2. Information on impacts
  - Impact description
  - Ecosystem component impacted (e.g. species, soil, etc.)
  - Magnitude of impact
  - Impact direction (negative, positive, neutral)
3. Additional information
  - Geographic region
  - Study Design (i.e. field or laboratory experiment, correlation or direct observation)
  - Notes

We will compile subsection *3c. Exceptions* on a case-by-case basis. For included studies, we will provide information by impact so that if a study investigated more than one, there will be a number of entries equivalent to the number of impacts assessed.

### Meta-data extraction

Studies included in the systematic literature map will undergo a full-manuscript screening to identify the investigated impact (or impacts). We will provide a description of the investigated impacts and the ecosystem component impacted. Then, we will categorize impacts by their magnitude and directionality. Impacts magnitude will be assessed following previous work, modified to include both positive and negative impacts [30–32]:

- Minimal: The impact is unlikely or negligible.
- Minor: It causes changes in the fitness of individuals in the native biota, but no changes in native population densities.
- Moderate: It causes changes in the population densities of native species, but no changes to the structure of communities or the abiotic or biotic components of ecosystems.
- Major: It causes the local or population extinction/introduction of at least one native species, and leads to reversible/transient changes in the structure of communities and the abiotic or biotic components of ecosystems.
- Massive: It leads to the replacement and local extinction/introduction of multiple native species, and produces irreversible changes in the structure of communities and the abiotic or biotic components of ecosystems.

### Synthesis and presentation

For each species, we will provide a first database with all studies included at the full-text screening and a reason for exclusions at this stage. A second database with the studies included in the map, along with a graphical representation of the screening process. Both databases will contain corresponding coded metadata (see *Data Coding* section). We will import studies included in the review into a reference manager and share them as a public library to facilitate accessibility. We will develop a graphical representation of riparian ecosystems, representing identified impacts and their magnitude and directionality for each species. Then, we will create a matrix combining multiple species (as rows) and impacts (as columns) to illustrate the collective impacts of the focus species. Descriptive statistics will be used to answer secondary questions. We will provide the geographic distribution of studies, visualize publication trends over time, and illustrate differences in species and impacts research effort. We will use co-occurrence matrices to identify research effort biases [63]. Lastly, we will provide a narrative synthesis of results for both main and secondary questions. The narrative synthesis will focus on (i) species and impact prioritization, (ii) clusters and gaps in present knowledge, (iii) predicted variations in impact magnitude and direction under current climate change scenarios, and (iv) avenues for future research.

## Supplementary Information

**Additional file 1.** List of primary studies selected for the comprehensiveness assessment of the searches.

**Additional file 2.** ROSES Checklist.

## Data Availability

Data sharing is not applicable to this article as no datasets were generated or analyzed during the current study.
