# Supplementary material for: Collating existing evidence on cumulative impacts of invasive plant species in riparian ecosystems of British Columbia, Canada: a systematic map protocol
Source: Environ Evid. 2023 Dec 14;12:31. doi: 10.1186/s13750-023-00320-3 (PMC11378858; doi:10.1186/s13750-023-00320-3)
Supplement: Supplementary file 1 — Additional file 1. List of primary studies selected for the comprehensiveness assessment of the searches. [file 13750_2023_320_MOESM1_ESM.docx]

**Appendix 1** – List of primary studies selected for the comprehensiveness assessment of the searches. Five studies meeting inclusion criteria were selected for each of the two pilot species utilized to develop the protocol.

***Reed canary grass (Phalaris arundinacea)***

1. Green E K & Galatowitsch S M. Effects of *Phalaris arundinacea* and nitrate-N addition on the establishment of wetland plant communities. Journal of Applied Ecology. 2002;39(1):134-144
2. Spyreas G, Wilm B W, Plocher A E, Ketzner D M, Matthews J W, Ellis J L & Heske E J. Biological consequences of invasion by reed canary grass (*Phalaris arundinacea*). Biological Invasions. 2010;12:1253–1267
3. Lesica P. Spread of *Phalaris arundinacea* adversely impacts the endangered plant *Howellia aquatilis*. The Great Basin Naturalist. 1997;57(4):366-368
4. Schooler S S, McEvoy P B, Coombs E M. Negative per capita effects of purple loosestrife and reed canary grass on plant diversity of wetland communities. Diversity and Distributions. 2006;12(4):351-363.
5. Wetzel P R & van der Valk A G. Effects of nutrient and soil moisture on competition between shape *Carex stricta*, shape *Phalaris arundinacea*, and shape *Typha latifolia*. Plant Ecology. 1998;138:179-190

**Russian Olive (*Elaeagnus angustifolia*)**

1. Follstad Shah J J, Harner M J, Tibbets T M. *Elaeagnus angustifolia* elevates soil inorganic nitrogen pools in riparian ecosystems. Ecosystems. 2010;13:46–61
2. Fischer R A, Valente J J, Guilfoyle M P, Kaller M D, Jackson S S, Ratti J T. Bird community response to vegetation cover and composition in riparian habitats dominated by Russian Olive (*Elaeagnus angustifolia*). Northwest Science. 2012;86(1):39-52
3. DeCant J P. Russian olive, *Elaeagnus angustifolia*, alters patterns in soil nitrogen pools along the Rio Grande River, New Mexico, USA. Wetlands. 2008;28:896–904
4. Katz G L, Tuttle G M, Denslow M W & Norton A P. Legacy effects of Russian Olive (*Elaeagnus* *angustifolia* L.) in a riparian ecosystem three years post-removal. Wetlands. 2020;40:1897–1907
5. Mineau M M, Baxter C V & Marcarelli A M. A non-native riparian tree (*Elaeagnus angustifolia*) changes nutrient dynamics in streams. Ecosystems. 2011;14:353–365
