# Supplementary material for: Collating existing evidence on cumulative impacts of invasive plant species in riparian ecosystems of British Columbia, Canada: a systematic map protocol
Source: Environ Evid. 2023 Dec 14;12:31. doi: 10.1186/s13750-023-00320-3 (PMC11378858; doi:10.1186/s13750-023-00320-3)
Supplement: Supplementary file 2 — Additional file 2. ROSES Checklist. [file 13750_2023_320_MOESM2_ESM.pdf]

Item	Section / sub-section	Topic	Description	Further explanation	Checklist/Meta-data	Author response	Comments
1	Title	Title	The title must indicate that it is a systematic map protocol, and must	The title should normally be the same or very similar to the review	Meta-data	Yes	
2	Type of review	Type of review	Select one of the following types of review: systematic map, systematic	See CEE Guidance on systematic mapping [1], and on amendments and	Meta-data	systematic map	
3	Authors contacts	Authors contacts	The full names, institutional addresses, and email addresses for all		Checklist	Yes	
4	Abstract	Structured summary	Abstract must not exceed 350 words and must include two sections 1)		Checklist	Yes	
5	Background	Background	Describe the rationale for the review in the context of what is already	A theory of change and/or conceptual model can be presented that links	Checklist	Yes	
6	Stakeholder engagement	Stakeholder engagement	The planned/actual role of stakeholders throughout the review process		Checklist	Yes	
7	Objective of the review	Objective	Describe the primary question and secondary questions (when	The primary question is the main question of the review. Secondary	Checklist	Yes	
8		Definitions of the question	Break down and summarise question key elements e.g. population,	For other question types see [4,5]	Meta-data	Yes	
	Methods						
9	Searches	Search strategy		Details regarding search strategy testing should be provided.	Checklist	Yes	
10		Search string	Provide Boolean-style full search string and state the platform for which		Meta-data	Yes	
11		Languages – bibliographic	List languages to be used in bibliographic database searches.		Meta-data	Yes	
12		Languages – grey literature	List languages to be used in organizational websites searches and web-		Meta-data	Yes	
13		Bibliographic databases	Provide the number of bibliographic databases to be searched.		Meta-data	Yes	
14		Web – based search engines	Provide the number of web – based search engines to be searched.		Meta-data	Yes	
15		Organisational websites	Provide the number of organisational websites to be searched.		Meta-data	Yes	
16		Estimating the	Describe the process by which the comprehensiveness of the search		Checklist	Yes	
17		Search update	Describe any plans to update the searches during the conduct of the	Optional. A search update is good practice if original searches were	Checklist	Yes	
18	Article screening and study	Screening strategy	Describe the methodology for screening articles/studies for		Checklist	Yes	
19		Consistency checking	Describe clearly the process for checking consistency of decisions		Checklist	Yes	
20		Inclusion criteria	Describe the inclusion criteria used to assess relevance of identified		Checklist	Yes	
21		Reasons for exclusion	State that you will provide a list of articles excluded at full text with		Checklist	Yes	
22	Critical appraisal	Critical appraisal strategy	Describe here the method you propose for critical appraisal of study	Optional	Checklist	No	
23		Critical appraisal used in	Describe how the information from critical appraisal will be used in	Optional	Checklist	No	
24		Consistency checking	Describe how repeatability of critical appraisal of study validity will be	Optional	Checklist	No	
25	Data extraction	Meta-data extraction and coding	Describe the method for meta-data extraction and coding for studies		Checklist	Yes	
26	Data synthesis and	Narrative synthesis strategy	Describe methods to be used for narratively synthesising the evidence	Vote-counting (tallying of studies based on the direction or significance	Checklist	Yes	
27		Knowledge gap and cluster	Describe the methods to be used to identify and/or prioritise key		Checklist	Yes	
28		Demonstrating procedural	Describe the role of systematic reviewers (who have also authored	Reviewers who have authored articles to be considered within the	Checklist	Yes	
29	Declarations	Competing interests	Describe of any financial or non-financial competing interests that the		Checklist	Yes	

References

[1] James, K.L., Randall, N.P. and Haddaway, N.R., 2016. A methodology for systematic mapping in environmental sciences. Environmental Evidence, 5(1), p.7.

[2] Bayliss, H.R., Haddaway, N.R., Eales, J., Frampton, G.K. and James, K.L., 2016. Updating and amending systematic reviews and systematic maps in environmental management. Environmental Evidence, 5(1), p.20.

[3] Haddaway, N.R., Kohl, C., da Silva, N.R., Schiemann, J., Spök, A., Stewart, R., Sweet, J.B. and Wilhelm, R., 2017. A framework for stakeholder engagement during systematic reviews and maps in environmental management. *Environmental Evidence* , *6* (1), p.11.

[4] Collaboration for Environmental Evidence. 2018. Guidelines and Standards for Evidence synthesis in Environmental Management. Version 5.0. www.environmentalevidence.org/information-for-authors.

[5] Leeds Institute of Health Sciences. https://medhealth.leeds.ac.uk/info/639/information_specialists/1500/search_concept_tools. Accessed 12/11/2017.
